# Enhanced Performance of Planar Perovskite Solar Cells Using Low-Temperature Solution-Processed Al-Doped SnO_2_ as Electron Transport Layers

**DOI:** 10.1186/s11671-017-1992-1

**Published:** 2017-03-31

**Authors:** Hao Chen, Detao Liu, Yafei Wang, Chenyun Wang, Ting Zhang, Peng Zhang, Hojjatollah Sarvari, Zhi Chen, Shibin Li

**Affiliations:** 1 State Key Laboratory of Electronic Thin Films and Integrated Devices, School of Optoelectronic Information, University of Electronic Science and Technology of China (UESTC), Chengdu, Sichuan 610054 China; 2 Department of Electrical and Computer Engineering, Center for Nanoscale Science and Engineering, University of Kentucky, Lexington, KY 40506 USA

**Keywords:** Perovskite solar cells, Electron transport layers, Low-temperature solution-process, Al-doped SnO_2_

## Abstract

Lead halide perovskite solar cells (PSCs) appear to be the ideal future candidate for photovoltaic applications owing to the rapid development in recent years. The electron transport layers (ETLs) prepared by low-temperature process are essential for widespread implementation and large-scale commercialization of PSCs. Here, we report an effective approach for producing planar PSCs with Al^3+^ doped SnO_2_ ETLs prepared by using a low-temperature solution-processed method. The Al dopant in SnO_2_ enhanced the charge transport behavior of planar PSCs and increased the current density of the devices, compared with the undoped SnO_2_ ETLs. Moreover, the enhanced electrical property also improved the fill factors (FF) and power conversion efficiency (PCE) of the solar cells. This study has indicated that the low-temperature solution-processed Al-SnO_2_ is a promising ETL for commercialization of planar PSCs.

## Background

The solar energy has attracted much attention since it is a renewable and clean energy source [1–4]. In recent years, a large amount of research groups have focused on organic-inorganic lead halide perovskite solar cells as it have the advantages of a lower manufacturing cost and a simpler process compared with Si solar cells. Moreover, PSCs have a great potential for providing an alternative to conventional photovoltaic devices. The PCE of PSCs has increased from 3.8 to 22.1% in a few years [5–9]. However, the efficiency and stability of PSCs strongly depend on some crucial factors, for instance, the morphology of perovskite films and the preparation of electron/hole transport material [10–15].

Both electron transport layers (ETLs) and hole transport layers (HTLs), which can extract electrons and holes from the light harvesting layers, respectively, are essential for the high-efficiency PSCs. Most of the high performance, PSCs were accomplished using compact TiO_2_ layer or mesoporous TiO_2_ layer as the ETLs [8, 16]. However, the processes of both the compact TiO_2_ layer and the mesoporous TiO_2_ layer generally require a high sintering temperature (>450 °C), which is an obstacle for the stretchable device fabrication and the commercial development of PSCs [17, 18]. Previously, SnO_2_ has shown up as an effective electron transport layer in perovskite solar cells due to the wider band gap (about 3.6 eV) and higher mobility (100 to 200 cm^2^V^−1^s^−1^) compared with TiO_2_ [19–21]. Furthermore, the temperature of forming SnO_2_ thin films (<200 °C) is helpful for widespread implementation and large-scale commercialization of PSCs [22]. Therefore, SnO_2_ is a promising candidate for ETLs used in high-performance PSCs.

It has been reported that doping ETLs with metal aliovalent cations are an effective method to improve properties of both ETLs and ETLs/perovskite interfaces. Other groups have already doped Y^3+^ and Li^+^ in SnO_2_ to improve carrier transport and optical abilities [23, 24]. In addition, doping can also improve the conductivity of ETLs and optimize the energy level matching between ETLs and the perovskite film, resulting in the enhanced performance of device [23].

Here, we present a low-temperature synthesized Al-doped SnO_2_ as an ETL in the n-i-p structure perovskite solar cells. Al-doped SnO_2_ thin films prepared at a low temperature (190 °C) show a better charge transport and electron extraction behavior than the pristine SnO_2_. The enhanced electrical property of SnO_2_ improves the PCE, *V*_OC_, *J*_SC_, and fill factor (FF) of the PSCs. The champion cell based on Al-doped SnO_2_ reaches a PCE of 12.10% with a *V*_OC_ of 1.03 V, a *J*_SC_ of 19.4 mA/cm^2^, and a FF of 58%, while the PSCs based on undoped SnO_2_ achieves a PCE of 9.02% with a *V*_OC_ of 1.00 V, a *J*_SC_ of 16.8 mA/cm^2^, and a FF of 53%.

## Methods

The fluorine-doped tin oxide (FTO) glass substrates were sequentially cleaned with acetone, ethyl alcohol, and deionized (DI) water in the ultrasonic bath for 15 min. Then the substrates were dried by a N_2_ flow and further cleaned by UV-ozone for 10 min before the deposition of SnO_2_.

SnO_2_ and Al-SnO_2_ ETLs were deposited by a spin-coating method. The solution was prepared by dissolving SnCl_4_•5H_2_O in isopropyl alcohol at a concentration of 0.075 M and subsequently stirred for 60 min at room temperature. For Al-doping, we dissolved AlCl_3_•6H_2_O in isopropyl alcohol. Then this aluminum precursor was added to the antecedent solution at a series of molar ratio and stirred until the solution became clear. Afterwards, the two different kinds of solution were separately deposited on cleaned FTO substrates at 3000 rpm for 30 s. The substrates were then pre-dried at 100 °C for 10 min and annealed at 190 °C for 1 h.

After the deposition of SnO_2_ and Al-SnO_2_ electron transport layers, the samples were treated by UV-ozone for 10 min again. The CH_3_NH_3_PbI_3_ film was fabricated by a one-step spin-coating method. The CH_3_NH_3_PbI_3_ solution (45 wt%) was deposited on the treated SnO_2_ at 5000 rpm for 30 s. 0.5 mL chlorobenzene was dropped onto the substrate when spin-coating the CH_3_NH_3_PbI_3_ solution. All the perovskite layers were annealed at 100 °C for 10 min. The hole transport layers were deposited by spin-coating the 2,29,7,79-tetrakis(N,Ndi-p-methoxyphenylamine)-9,9-spirobifluorene (spiro-OMeTAD) solution at 4000 rpm for 30 s. Finally, 100-nm-thick gold top electrode was deposited on the HTL via thermal evaporation.

### Device Characterization

The J-V characteristics of perovskite solar cells were measured by Keithley 2400 source measuring unit under the AM 1.5 G solar-simulated light (Newport Oriel Solar 3 A Class AAA, 64023 A). The sun light (100 mw/cm^2^) was calibrated by a standard Si-solar cell (Oriel, VLSI standards). X-ray photoelectron spectroscopy (XPS) was measured using the Kratos XSAM 800 X-Ray Photoelectron Spectrometer.

## Result and Discussion

**Fig. 1:**
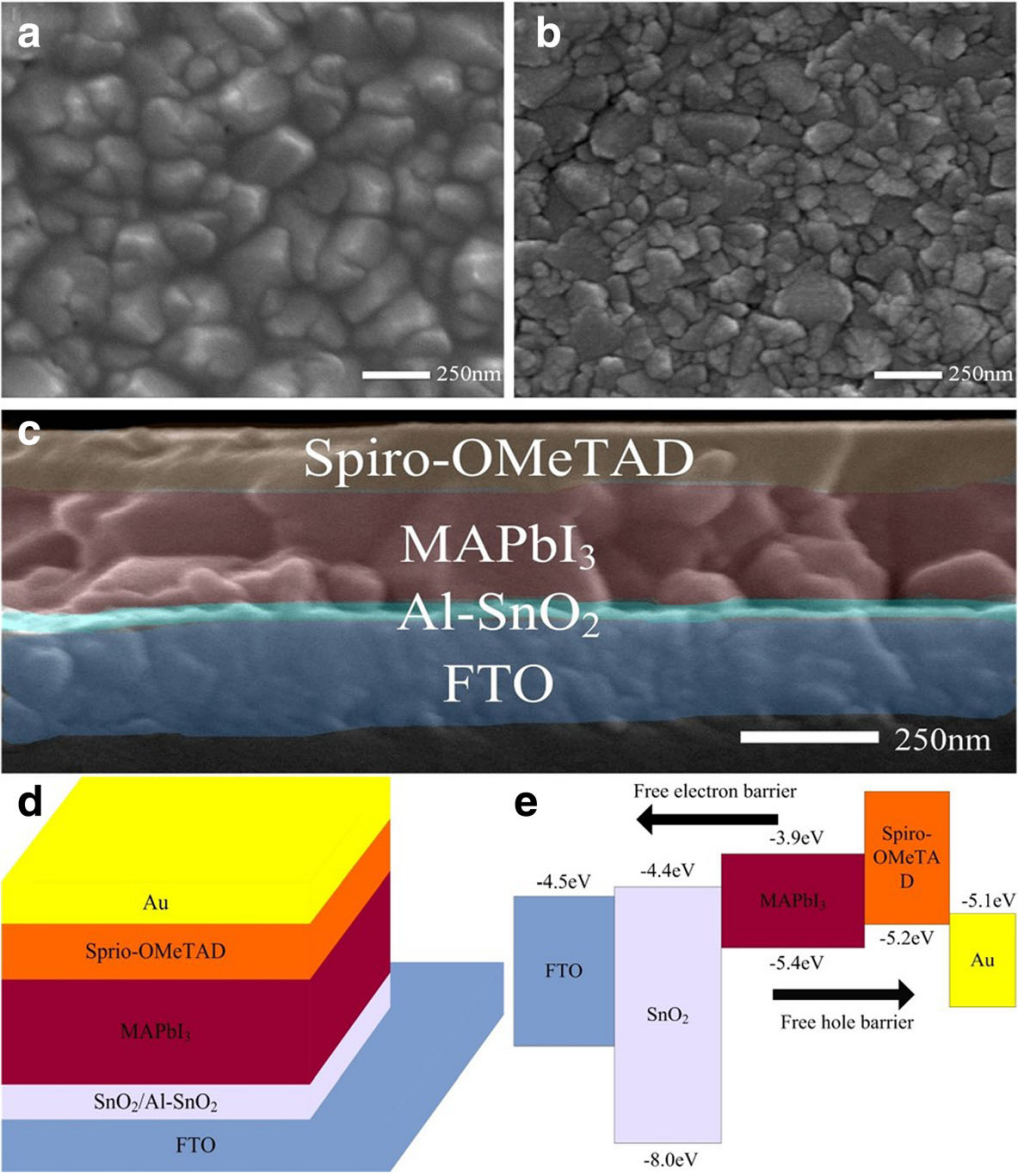
**a** Top view SEM images of low-temperature solution-processed SnO_2_ on FTO and **b** bare FTO. **c** Cross-sectional SEM image of the planar perovskite solar cell based on Al-SnO_2_, **d** schematic of the PSCs grown on Al-SnO_2_, and **e** energy band diagram of the PSCs

In our work, SnO_2_ layers have been deposited on FTO substrates by a low-temperature solution method. Top view scanning electron micrographs (SEM) of the SnO_2_ and bare FTO are shown in Fig. 1a–b. A dense and pinhole-free film is formed by spin coating SnO_2_ ETLs solution on FTO substrates, suggesting that the FTO substrates have been fully covered. Dense ETLs are known as an essential element of high-performance PSCs. Thus, a compact SnO_2_ layer deposited on FTO can enhance the interfacial contact with perovskite layers and improve the performance of the solar cells [25].

To examine the efficiency of PSCs based on the low-temperature solution-processed SnO_2_ and Al-SnO_2_ ETLs, we have fabricated the planar solar cells with the structure of FTO/(Al-)SnO_2_/MAPbI_3_/Spiro-OMeTAD/Au shown in Fig. 1d. In addition, Fig. 1c shows a cross-sectional SEM image of a PSC based on Al-doped SnO_2_ layer without the Au electrode, and the energy band diagram of PSCs is shown in Fig. 1e.

**Fig. 2:**
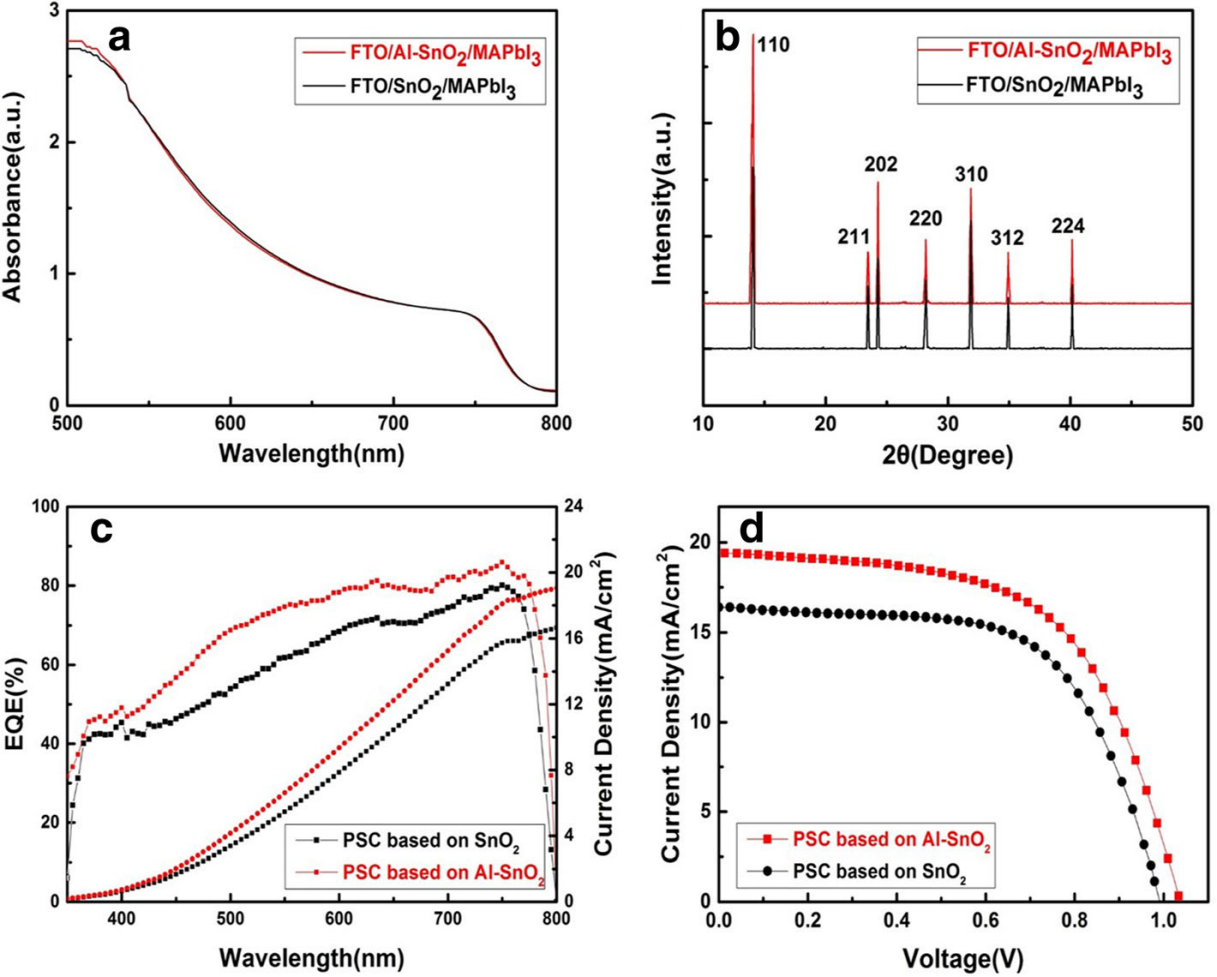
**a** UV-vis absorbance spectra and **b** XRD patterns of perovskite films grown on SnO2 and Al-SnO2 substrates. **c** EQE curves and integrated current density of perovskite solar cells based on SnO2/Al-SnO2 and **d** J-V curves of the best-performing PSC using SnO2/Al-SnO2 ETLs

To confirm that the employment of Al-doping SnO_2_ as ETLs has no effect on the perovskite films, we measured the UV-vis absorbance spectra and the corresponding X-ray diffraction (XRD) of the MAPbI_3_ film on FTO/SnO_2_ and FTO/Al-SnO_2_ substrates. The results are shown in Fig. 2a and b, respectively. The MAPbI_3_ films deposited on SnO_2_ and Al-SnO_2_ ETLs exhibit almost identical absorbance spectra, suggesting that the absorption of the perovskite films is nearly not affected by Al-doping in SnO_2_ ETLs.

As to the XRD patterns, several strong peaks are located at 14.05, 23.44, 24.25, 28.18, 31.88, 34.93, and 40.16°. All these peaks can be assigned to orthorhombic crystal of the perovskite with high crystallization [26–28]. The XRD patterns show negligible difference between the samples of FTO/SnO_2_/MAPbI_3_ and FTO/Al-SnO_2_/MAPbI_3_, indicating the dopant of Al in SnO_2_ film does not affect on the structure property of MAPbI_3_ film. Furthermore, the main PbI_2_ peak is absent from the XRD patterns, which indicates PbI_2_ has sufficiently reacted with MAI.

As the evidences were obtained from the UV-vis absorbance spectra and XRD patterns of the devices, the dopant of Al in SnO_2_ does not cause any obvious changes on the structure and optical properties in the perovskite layers. Therefore, the performance enhancement induced by Al-doping in SnO_2_ as ETLs is most likely due to the improvement of the ETLs/perovskite interfacial properties. In other words, the charge transport and electron extraction are improved.

Figure 2c illustrates the external quantum efficiency (EQE) spectrum of the best-performance solar cells based on SnO_2_ and Al-SnO_2_. Obviously, the EQE of Al-doped device is higher than the device based on pristine SnO_2_ over the entire wavelength range. The higher EQE means superior electron extraction capability of the ETLs [29]. The calculated *J*_SC_ (≈19.0 mA/cm^2^) based on Al-SnO_2_ from the EQE spectra is consistent with the measured value of the current density-voltage (J-V) curves measured under the one-sun light. As for undoped SnO_2_, the calculated *J*_SC_ is approximately equal to 16.6 mA/cm^2^.

The J-V curves of the best-performance PSCs based on SnO_2_ and Al-SnO_2_ ETLs are shown in Fig. 2d. The PCE increases from 9.02 to 12.10% by doping SnO_2_ with Al. Al-doping may cause an improvement on the charge transport and electron extraction behavior of the SnO_2_, leading to the increment of the *J*_SC_ (16.8 to 19.4 mA/cm^2^). Furthermore, the *V*_OC_ of the best PSC based on Al-SnO_2_ (1.03 V) is a little higher than that of the best cell based on SnO_2_ (1.00 V), indicating less energy loss of electrons [30]. Therefore, the enhanced parameters mentioned above leads to the improvement of FF (53 to 58%).

**Fig. 3:**
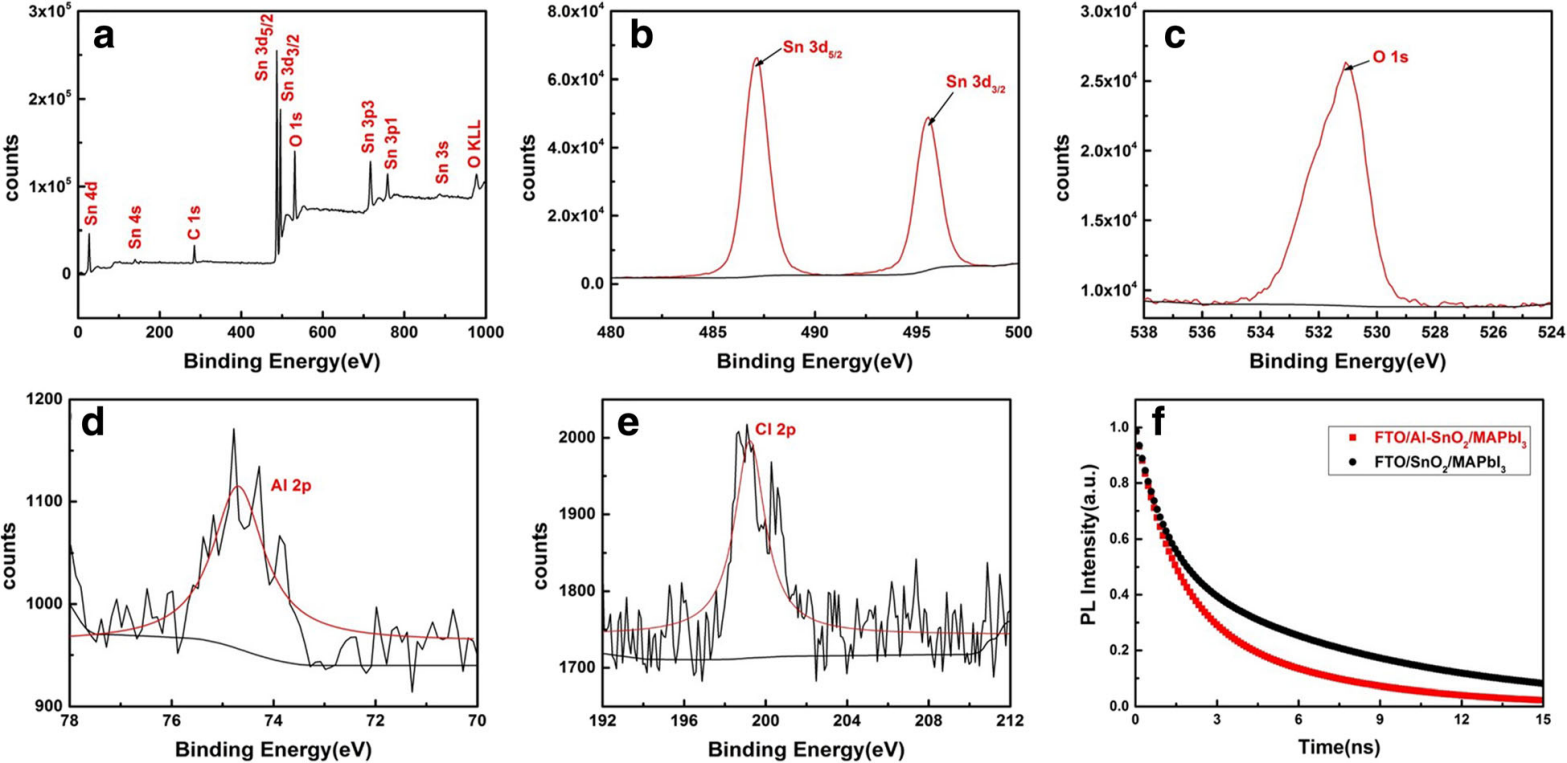
XPS spectra of **a** survey, **b** Sn 3d, **c** O 1 s, **d** Al 2p, and **e** Cl 2p peaks for Al-SnO_2_ film deposited on the FTO substrate. **f** Normalized time-resolved PL decay curves of MAPbI_3_ films deposited on SnO_2_ (*black line*) and Al-SnO_2_ (*red line*) coated substrates

Al-SnO_2_ films deposited by a low-temperature solution-processed were further investigated by X-ray photoemission spectroscopy (XPS). Figure 3a displays the full XPS spectrum, which shows the presence of O, C, and Sn. The binding energies of 487.3 and 495.8 eV shown in Fig. 3b corresponds to Sn 3d_5/2_ and Sn 3d_3/2_, respectively. The main binding energy of 531.0 eV shown in Fig. 3c corresponds to O 1 s, which reveals the O^2−^ state in SnO_2_ [21]. The absence of Al in the full XPS spectrum can be attributed to the low concentration (5% molar ratio), while Al 2p peak can be observed in Fig. 3d with a relatively low content, indicating that the doping is truly practicable. Identically, the Cl 2p peak missed in the full XPS spectrum can also be observed in Fig. 2e. The low-content Cl suggests that both most of SnCl_4_ and AlCl_3_ have been oxidized.

To identify the reasons for the enhanced performance due to the Al-doping, we carried out the time-resolved photoluminescence (TRPL) to study the electron-extraction behavior of different ETLs. The TRPL decay curves, shown in Fig. 3f, are exponentially fitted, where τ1and τ2 represent the bulk recombination in perovskite bulk films and the delayed recombination of trapped charges, respectively [31]. For the FTO/SnO_2_/MAPbI_3_ sample, τ1 is 1.07 ns and τ2 is 7.98 ns, while τ1 is 1.32 ns and τ2 is 5.13 ns for the doped SnO_2_ sample (1% Al-doping content). Apparently, the perovskite film deposited on the Al-SnO_2_ ETL has a lower τ2 with a lower ratio of τ2/τ1, indicating a better change transfer from perovskite to ETLs and more efficient extraction of the photo-induced electrons between the perovskite and ETLs, as compared to the film deposited on the pristine SnO_2_ ETL [32, 33]. In addition, the decay curves also confirm the remarkable enhancement of the electron extraction and charge transport induced by Al-doping in SnO_2_. These properties result in the improvement of current density and the power conversion efficiency.

**Fig. 4:**
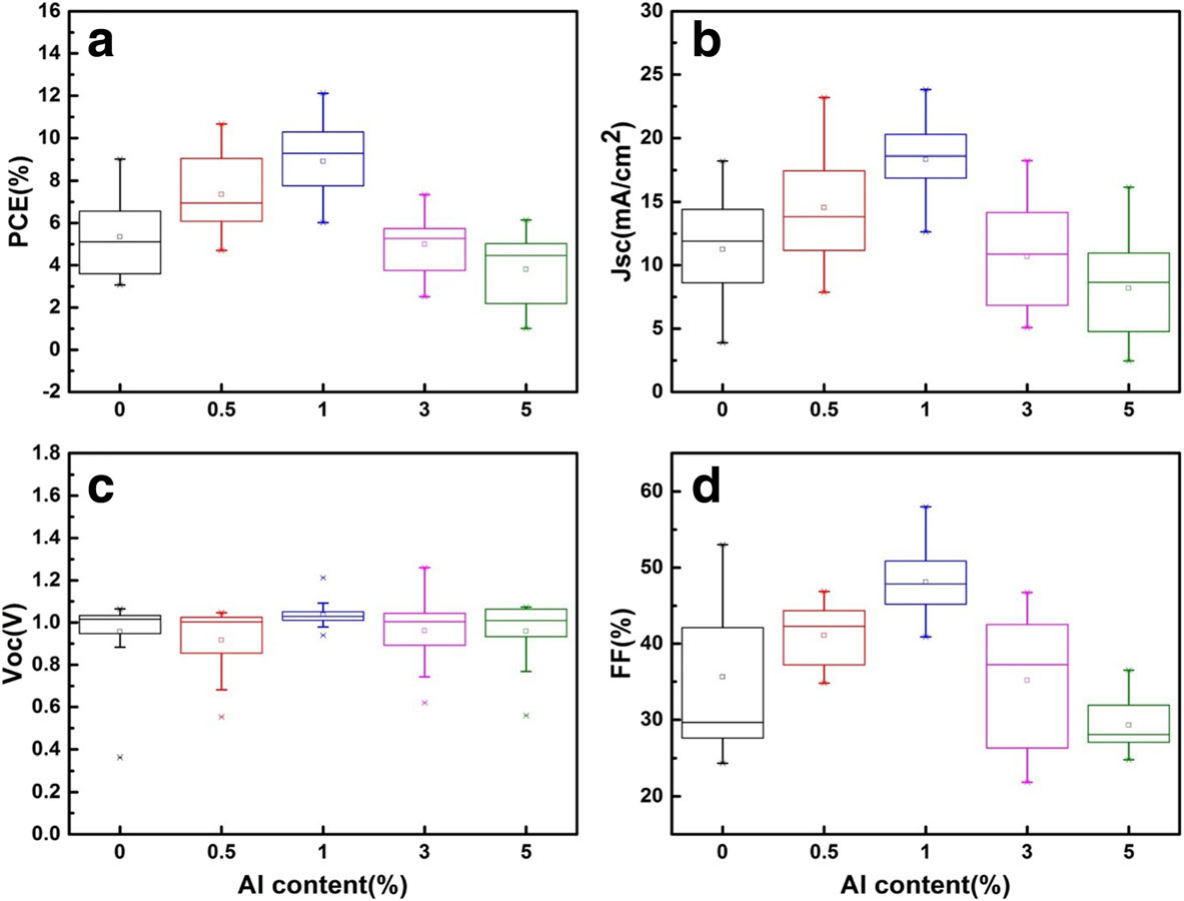
Histograms of the different Al content on photovoltaic parameters including **a** PCE, **b** J_SC_, **c** V_OC_, and **d** FF

We also compared four different parameters of the cell performance with a series of doping concentration. From the box charts in Fig. 4a, it is obvious that the PCE of the cells is strongly influenced by Al doping. The average PCE of the cells is improved with the increment of Al content before the concentration of 1%, while the average PCE is reduced with the higher Al content (3 and 5%). The change of *J*_SC_ of these solar cells is shown in Fig. 4b, and the variation tendency is like the trend of PCE. The highest *J*_SC_ is 23.82 mA/cm^2^, which confirms a good charge transportation of the cells. Regarding the change of *V*_OC_, Fig. 4c shows the variation of *V*_OC_, value is smallest with 1% Al-doping content. The results demonstrate that the solar cells with 1% Al-doping exhibit the best repeatability. As exhibited in Fig. 4d, the change tendency of FF is analogous to the trend of PCE. In addition, the average FF of the solar cells doped with 0.5 and 1% Al^3+^ content is higher than the undoped solar cells impressively. The results mentioned above reveal that a suitable Al-doping is beneficial for the performance of the perovskite solar cells based on SnO_2_.

## Conclusions

In summary, we studied the effect of Al-doping on SnO_2_ as ETLs for planar perovskite solar cells. According to the results of UV-vis absorbance spectra and XRD patterns of perovskite films deposited on Al-SnO_2_ and SnO_2_, the Al dopant in SnO_2_ does not influence the structure and optical properties of the perovskite layers. Based on the TRPL test, the Al-dopant in SnO_2_ enhances the charge transport and electron extraction behavior of the PSCs and then the *J*_SC_ of the devices is improved. The champion cell based on Al-SnO_2_ exhibited a higher efficiency of 12.10% than that using SnO_2_ (9.02%) as ETLs. Our results suggest that efficient planar perovskite solar cells based on SnO_2_ can be fabricated by doping SnO_2_ with Al^3+^.
